# The impact of early life antibiotic use on atopic and metabolic disorders: Meta-analyses of recent insights

**DOI:** 10.1093/emph/eoaa039

**Published:** 2020-10-24

**Authors:** Semeh Bejaoui, Michael Poulsen

**Affiliations:** Section for Ecology and Evolution, Department of Biology, University of Copenhagen, Universitetsparken 15, 2100 Copenhagen East, Denmark

**Keywords:** atopies, coevolution, dysbiosis, immune systems, metabolism, microbiome

## Abstract

**Background and objectives:**

The impact of antibiotics use early in life on later-in-life morbidities has received substantial attention as explanations for atopic and metabolic disorders with a surge as modern lifestyle diseases. The objective of this study was to perform meta-analyses to determine if antibiotics administration during the first 2 years of infant life is associated with increased risks of atopic or metabolic disorders later in life.

**Methodology:**

We screened more than 100 English-language prospective and retrospective studies published between January 2002 and March 2020 and assessed study quality using the Newcastle–Ottawa scale. We performed overall and subgroup meta-analyses on 31 high-quality comparable studies on atopic and 23 on metabolic disorders, involving more than 3.5 million children.

**Results:**

Antibiotic exposure prenatally and during the first 2 years of life significantly impacts the risk of developing atopic and metabolic disorders. Exposure during the first 6 months of life appears most critical, consistent with this being the time when the microbiome is most susceptible to irreversible perturbations. The presence of dose−response associations and stronger impacts of broad- than narrow-spectrum antibiotics further point to effects being mediated by microbiota-induced changes.

**Conclusions and implications:**

Our findings support that antibiotics use is a mismatch to modernity that can negatively affect the symbiotic associations we rely on for proper immune function and metabolism. Improving our understanding of these associations, the underlying proximate mechanisms and the impact of antibiotics use on future human−symbiont evolution will be important to improve human health.

**Lay Summary:**

The use of antibiotics in infancy has been suggested to increase the risks of atopic and metabolic disorders later in life. Through meta-analyses of more than 100 studies of >3.5 million children, we confirm these risks, and show that patterns are consistent with effects being due to microbiota-driven changes.

## INTRODUCTION

Humans have a long evolutionary history of associations with gut microbiome members. These interactions are in many cases beneficial, supporting essential functions in digestion, immunity and cognition, and microbial communities consequently influence our development, growth and health [1–3]. In return, the bacteria benefit from the stable gut environment and nutrient provisioning [4]. The advantages conferred to the host are the result of a finely tuned relationship between the individuals and a healthy microbiota, which is not characterized by having a fixed ‘health-promoting’ structure, but rather by the presence of a diverse, stable and resilient community within which competition and cooperation have overall positive effects on the human host [5]. The composition of these communities is non-random and have substantial consequences for human health, often through factors that are still poorly understood [6, 7]. 

**Figure 1.**
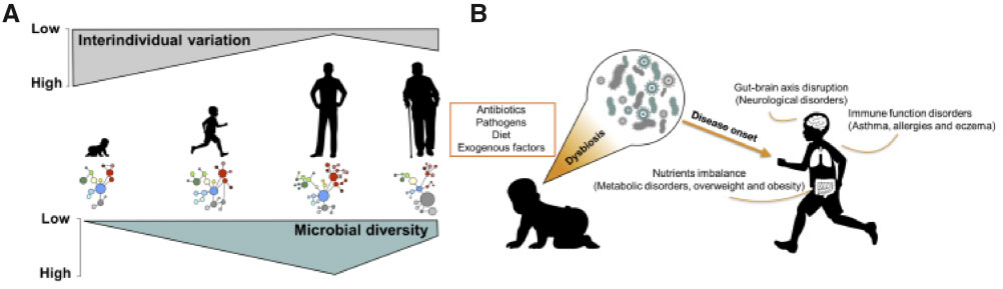
The process of normal establishment of a healthy microbiota (**A**) and potential consequences of early onset dysbiosis (**B**). The microbiota of infants starts as less diverse and with high inter-individual variability; upon reaching adulthood the microbiota stabilizes, presenting increased diversity and more similarities in composition between individuals. The introduction of early disturbers such as antibiotic use or pathogen infection during development can impact the infant microbiota and lead to dysbiosis, which ultimately may lead to a series of metabolic or atopic disorders.

The infant microbiota starts as simple and unstable in composition and undergoes substantial fluctuations that result in its progressive enrichment and stabilization (Fig. 1A). Colonization of the infant intestinal tract begins at birth [8], and initial interactions between microbes and the neonatal mucosal and immune system set the stage for development of the microbiome with life-long consequences [9]. Because the immune system of newborns is immature and with high tolerance to external stimuli, microbes can establish without triggering inflammation, and these colonizers are later recognized as beneficial [10]. By the end of the first year of life, infant microbiomes acquire more adult-like characteristics and by ∼3 years of age, although still prone to modifications, typically resemble adult composition and diversity [7, 11] (Fig. 1A). Due to its naïve form, the infant gut microbiota is prone to perturbations by exogenous factors, which can impair the establishment and proliferation of the appropriate bacteria [12]. Microbiota development can thus be affected by delivery mode, diet, genetics, health status, gestational age and antibiotic use [13–17]. Improper development can have negative consequences for metabolism, immune and brain functions, and may even play roles in behavioral or neurodevelopmental disorders [7, 18, 19].

Of the exogeneous factors that can impair the developing microbiomes, use of antibiotics can have both short- and long-term negative effects. Most of the antibiotics used are broad spectrum and thus do not discriminate between pathogenic and beneficial microorganisms; this can potentially impact beneficial community structure and ultimately lead to dysbiosis [20, 21]. One major consequence of losing important symbionts is the disruption of immune homeostasis and the alteration in the mucosal immune response, which can predispose to enteric infections and increase levels of inflammation [22, 23]. Worldwide, antibiotics are the most commonly prescribed medicine given to children to treat infections [24]. In Europe and the USA, ∼1% of children receive at least one course of antibiotics before age 2 years, while children in Asia receive up to three times as many courses during this early period of life [25]. In lower-income countries, the situation is even more striking: during the first 2 years of life, children in India, Pakistan, Brazil or South Africa are exposed to antibiotics courses corresponding to more than 4 months of cumulative treatment [26]. Up to 90% of these antibiotics are available without prescription [27].

The use of antimicrobials in children may not only have short-term effects, but also later impact morbidity, representing a potentially severe mismatch to modernity in contemporary human societies. In this context, the links between antibiotics use early in life and later-in-life consequences has received substantial attention for two major groups of disorders that have seen a surge as modern lifestyle diseases: atopic and metabolic disorders. Many studies point toward the first months of life as a critical window during which the infant gut microbiota influences key immunologic events, and where antibiotics administration may alter allergenic sensitization and potentially affect the development of asthma and atopies [28–30]. Similarly, antibiotics-induced reduction in microbial diversity and depletion of beneficial bacteria may lead to increased body mass index (BMI) and increased risks of obesity and its comorbidities. Disruption may result in an ‘obesogenic’ microbiota, characterized by reduced diversity and an imbalance in bacterial genes involved in metabolite production; eventually, the increased levels of fatty acids—the increased energy extracted from the food—contribute to excessive weight gain [31–34]. Expanding our understanding of these associations is necessary to improve public health but also to grasp all the fundamental evolutionary underpinnings of the impact of antibiotics use on human symbionts and how they in turn may affect our evolution.

A number of studies has tried to disentangle these relationships: exposure to at least one course of antibiotics within the first 2 years of life appears to be a risk factor for the development of childhood asthma, atopic dermatitis and atopies [35–37]. Conversely, a similar analysis reported a weaker association between asthma development and antibiotic exposure during the same period of time [38]. For metabolic disorders, Shao *et al.* [39] showed that antibiotic exposure early in life significantly increases the risk for childhood adiposity, similar to a more recent meta-analysis of 23 cohort studies [40]. Antibiotic exposure was associated with a slightly increased risk of overweight or obesity in children exposed during the first 6 months of life, but only after repeated treatments [41]; similarly, a small increase in the risks of childhood overweight and obesity was observed with exposure before 2 years of age, but only in small subgroups of children [42]. Among the studies available, the majority suggests a correlation; at the same time, however, a similar number of studies challenges these conclusions, either rejecting the significance of the association or circumscribing it to specific risk categories. Since reaching univocal conclusions has proven challenging, the objective of this study was to perform more comprehensive systematic meta-analyses to determine if antibiotics administration during the first years of infant lives is associated with increased risks of atopic and metabolic disorders later in life.

## METHODS

### Literature searches

We searched the PubMed and PMC databases for all relevant studies published up to March 2020. For atopic disorders, we examined the impact of prenatal or early life antibiotic exposure on later asthma, eczema and allergies development. The search terms used were: (child OR children OR childhood OR early life OR early childhood OR infancy OR infants) AND (asthma OR allerg* OR atop* OR wheeze OR eczema) AND (antibiotic OR antibiotics OR antimicrobial OR antimicrobials). For metabolic disorders, we examined the impact of prenatal or early life antibiotic exposure on childhood adiposity. The search terms used were: (child OR children OR childhood OR infancy OR infants) AND (antibiotic OR antibiotics OR antimicrobial OR antimicrobials) AND (adiposity OR obesity OR obese OR overweight OR body mass index). Additional screening was manually performed on bibliographies of eligible articles and relevant reviews.

### Study selection for meta-analyses

Studies on atopies were included in the meta-analysis if they met the following inclusion criteria: (i) they were on the topic of association between antibiotic exposure and the risk of childhood asthma, eczema or allergies; (ii) they were prospective or retrospective cohort studies; (iii) the exposure time was prenatal period and infancy, generally defined as the period from birth to 2 years of age; (iv) they reported odds ratios (ORs) with 95% confidence intervals (CIs) as risk estimates, or relative risks that we converted to ORs. Similarly, studies on metabolic disorders were included in the meta-analysis if they met the following inclusion criteria: (i) they were on the topic of association between antibiotic exposure and the risk of childhood overweight, obesity or increased BMI; (ii) they were prospective or retrospective cohort studies; (iii) the exposure time was prenatal period and infancy, generally defined as the period from birth to 2 years of age; (iv) they reported ORs or mean difference (MD) with 95% CI as risk estimate, or relative risks that were converted to ORs.

### Data extraction and quality assessment

For both categories of studies, we independently extracted relevant data from all included articles. Disagreements or discrepancies on extracted data were resolved by discussion and consensus between the authors. The extracted data included first author, country and baseline time, number of participants, study design, exposure period, follow-up time, outcome of interest and adjusted variables. Studies were also assessed for their quality using the Newcastle–Ottawa scale (NOS) [43], which evaluates the risk of bias of observational studies: the scale divides the studies based on selection and comparability of the study groups, and ascertainment of the outcome of interest, and awards a maximum total of nine points; studies scoring 7 or more are generally considered high quality [44, 45].

### Statistical analyses

Studies were first divided by their outcome (asthma, eczema or food/non-food allergies for the analysis on atopies and obesity, overweight or BMI-*z* score for metabolic disorders) before performing the analyses. The risk estimate of association between antibiotics exposure and the dependent variables was calculated by pooling the single ORs [or MDs for studies reporting the difference in age- and sex-standardized BMI score (BMI-*z*)] and their respective 95% CIs. Statistical heterogeneity among studies was assessed using the *I*^2^ method [46], and a random-effect model was used on pooled data to reduce the impact of heterogeneity. For studies providing more than one effect size, data consistent with our criteria were included with a random effect, clearly specifying the different effects. *P*-values below 0.05 were considered statistically significant. We assessed the presence of publication bias by visually evaluating the symmetry of the corresponding funnel plots, where symmetric plots suggest that a bias is unlikely. All statistical analyses were carried out using the Review Manager (5.4 version) software and the ‘meta’ package [47, 48] in R [49].

## RESULTS

### Literature searches

The initial literature search produced a total of 1442 abstracts on atopic disorders, 1352 of which were excluded for being irrelevant to the topic or in a language other than English. Twenty-three studies were further removed for being on animals, reviews, other meta-analyses or commentaries. The remaining 67 studies were assessed by reading their full-texts, after which 36 were excluded for being case−control studies, making them hard to compare to cohort studies, scoried low on the NOS, or examined outcomes or exposure times that were inconsistent with our criteria. Ultimately, 31 cohort studies including 2,488,097 participants were included [30, 50–79].

For metabolic disorders, the initial literature search produced a total of 1228 abstracts, of which 1197 were excluded for being of irrelevant topic or in another language. Thirty-three studies were further excluded for being reviews, commentaries or meta-analyses. The remaining 35 studies were assessed by reading their full texts and 12 studies were consequently excluded because they did not match our inclusion criteria. Ultimately, 23 cohort studies including 1,196,962 participants remained [32, 80–101].

### Characteristics of included studies

The main characteristics of the included studies on atopic disorders are summarized in Supplementary Table S1. There were 20 prospective [30, 50–52, 57–61, 63–65, 69, 70, 72, 74–78] and 11 retrospective [53–56, 62, 67, 68, 71, 73, 79] studies with publication periods ranging from 2005 to 2020 and baseline study periods ranging from 1959 to 2018. The number of participants varied greatly, from 198 [61] to 792,130 [68] children. Participants were followed for an average of 6 years, generally with a single follow-up time point, but nine studies had follow-ups for several years [52–56, 59, 63, 67, 69]. Most studies (11) were from the USA, followed by 4 from Canada, 2 from Japan and the remaining from Europe. One study collected data from 29 different countries (Supplementary Table S1). Time of antibiotics exposure varied, with 7 studies examining the prenatal period, 4 the first 6 months of life, 14 the first year of life, and 6 the first 2 years of life (Supplementary Table S1). The majority of studies (28 out of 31) investigated the relationship between antibiotic exposure and asthma development, while only seven studies considered the food or non-food allergies and nine examined the development of eczema. All studies reported risk estimates and adjusted risk estimates, but although the investigated confounding factors differed, all studies included maternal history factors, which is known to be important [102]. The quality of studies ranged from 7 to 9 on the NOS scale (average 7.9), suggesting overall high methodological quality (Supplementary Table S1).

The main characteristics of the included studies on metabolic disorders are summarized in Supplementary Table S2. Fifteen studies were prospective and eight were retrospective, with publication periods from 2011 to 2020 and baseline study periods from 1959 to 2016. The number of participants ranged from 97 [100] to 333,653 [98] children, who were followed for an average of 7 years, generally with follow-up at a single time point. All studies took place in Europe or North America, 13 were in the USA, 2 in the UK, and the rest in Denmark (4), Finland (1) and The Netherlands (1). One study included 18 countries. Exposure timing varied: six studies examined the prenatal period, four the first 6 months of life, six the first year of life and seven the first 2 years of life. Ten of the 23 studies investigated the risk of obesity, six considered the risk of becoming overweight and the remaining evaluated BMI-*z* score. All studies reported risk estimates and adjusted risk estimates, and their NOS values ranged from 7 to 9 (average 8.1), suggesting overall high quality (Supplementary Table S2).

### Atopic disorders

**Figure 2.**
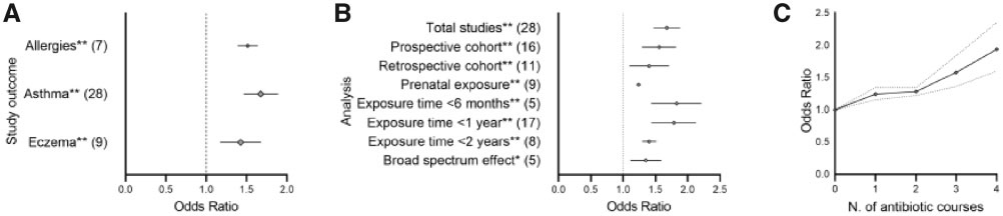
Main findings in overall and subgroup meta-analysis of immune-related studies given as pooled ORs with 95% CI; for the full results, see Supplementary Table S1. (**A**) Pooled OR risks for all three immune-related disorders across all available studies. Significance levels indicated with asterisks: **P < 0.0001. Dashed line indicates OR of 1; i.e. no difference in risk to controls. (**B**) Asthma studies and their outcomes (OR) with total studies and sub-analyses. The number of studies considered are indicated in brackets. Significance levels after post hoc analyses indicated with asterisks: *P = 0.01 and **P < 0.0001. Dashed line indicates OR of 1; i.e. no difference in risk to controls. (**C**) Dose−response meta-analysis of the association between early antibiotic exposure and childhood asthma (*n* = 6). Solid and dashed lines represent estimated pooled ORs and CIs, respectively.

In a random effect model, the overall pooled results showed a positive association between the early administration of antibiotics and the risk of developing either asthma, eczema or allergies (Fig. 2A). The larger number of studies investigating the risk of developing asthma allowed us to perform subgroup analyses to evaluate effects of exposure timing, study design and antibiotics type, which reduced study heterogeneity (Supplementary Table S3), but associations remained positive (Fig. 2B). The period from birth to 6 months of age was when infants are most vulnerable; with reduced risks of effects during 6 − 12 and 12 − 24 months (Fig. 2B). Furthermore, and as predicted from studies pointing to increased risks associated with broad-spectrum antibiotics, we found a stronger effect of broad- than narrow-spectrum antibiotics (Fig. 2B); however, notably with substantial heterogeneity between studies (92%). The comparison of study design showed that prospective and retrospective cohort studies can be considered comparable (Fig. 2B;  Supplementary Table S3), and lastly, we found a significant positive relationship between the number of antibiotics courses and the risk of developing childhood asthma (Fig. 2C). There were insufficient studies to perform sensitivity analysis for eczema and allergies; however, the latter showed no obvious heterogeneity (Supplementary Table S3; for individual sensitivity analyses, see Supplementary Fig. S1).

### Metabolic disorders

**Figure 3.**
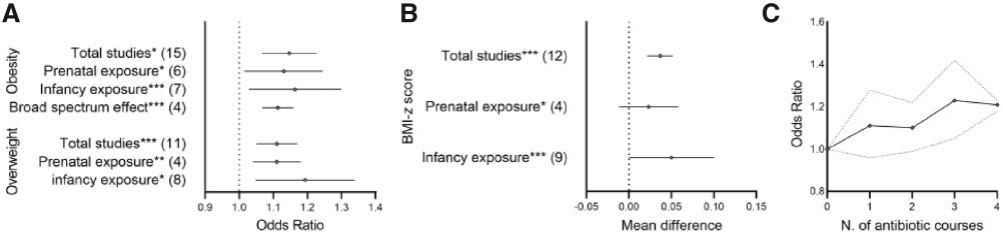
Main findings in overall and subgroup meta-analysis of metabolic-related studies. For the full results, see Supplementary Table S2. (**A**) Obesity and overweight studies and their outcomes (OR, 95% CI). The number of studies included are given in brackets. Significance levels are indicated with asterisks: *P < 0.05, **P < 0.001 and ***P < 0.00001. Dashed line indicates OR of 1; i.e. no difference in risk to controls. (**B**) Meta-analysis of BMI-*z* score studies represented as main difference (MD***) and 95% CI. Significance levels are indicated with asterisks: *P < 0.05 and ***P < 0.00001. Dashed line at MD = 0 indicates the point at which there is no difference to controls. (**C**) Dose−response meta-analysis of the association between early antibiotic exposure and childhood obesity (*n* = 4). Solid and dashed lines represent the estimated pooled ORs and CIs, respectively.

In a random effect model, all analyses showed a small but positive and significant association between antibiotic exposure and the risk of developing childhood adiposity, although with substantial heterogeneity across studies (Fig. 3A;  Supplementary Table S4). Sensitivity analyses were therefore performed for each outcome, with the subgroups considered being prenatal vs infancy (0 − 24 months) exposure, and broad- vs narrow-spectrum antibiotics (Supplementary Table S4). Results from these sub-analysis confirmed the overall positive association but a larger impact of exposure in infancy than prenatally (Fig. 3B). Exposure to broad-spectrum antibiotics significantly increased the risk of developing childhood obesity than narrow-spectrum antibiotics exposure (Fig. 3A;  Supplementary Table S4), but although this trend was positive across the four studies that could be included for this test, we did not find a significant dose−response effect on the increased risk of childhood obesity (Fig. 3C). Individual sensitivity analyses are provided in Supplementary Figs S2 and S3.

## DISCUSSION

### The novelty of our findings: statistical confirmation of ambiguous associations

Human behavior has changed dramatically over our evolutionary historym and modern living is characterized by a richer diet, a more sedentary lifestyle, and easy access to antibiotics and other drugs. All of these have affected not only us but also our microbiota, with consequences for microbiota-dependent functions, such as digestion and immunological tolerance [103]. Different studies over time have sought to evaluate whether these effects are exacerbated if antibiotics exposure occurs prenatally or in the very early stages of infancy; i.e. during the period of development and stabilization of the infant microbiota (Fig. 1A), but results remained conflicting. Our screening of a large body of recent literature and meta-analyses of comparable studies allowed for a rigorous evaluation of the proposed effects of early antibiotics exposure to later-in-life atopic and metabolic disorders. In the largest meta-analysis to date, our general and subgroup analyses including more than 3.5 million children support that antibiotic exposure can significantly impact the risk of developing these disorders. Although our findings remain correlational, they are consistent with microbiota-driven effects, because we observe stronger effects in children with more naïve and vulnerable (i.e. <6 months of age) than more established (1 − 2 years of age [11]) microbiomes, effects of the number of doses (significant for asthma) and stronger effects of broad- than narrow-spectrum antibiotics, consistent with their more profound effects on commensal microbes. Consistent with this, prenatal exposure appears less critical than exposure during infancy, likely because of reduced direct impact on the microbiota of the children, while effects are conceivably due to modifications of the more resilient maternal vaginal and placental microbiota.

Our analyses are consistent with previous smaller meta-analyses, reporting impacts of antibiotic use on autoimmune and metabolic disorders [35–37, 39, 40]. However, discrepancy observed with other analyses, such as Penders *et al.* [38], can be explained by the fact that different studies include different confounding factors, which can lead to an over- or underestimation of effects. If the same restriction criteria were always applied to all meta-analyses, similar conclusions would be more likely, although the high variability among studies would preclude complete extrapolation. Indeed, the heterogeneity of the studies, in terms of different confounding factors considered and the biases they introduce, is one of the major limitations of meta-analyses. For this reason, performing sensitivity sub-analyses is useful to gain deeper insights into the patterns of associations across datasets, but doing so inevitably at the same time reduces sample sizes and analyses power. Nonetheless, our study has significant added value by including the largest number of studies (to our knowledge) to date, thus maintaining high power. Moreover, this is (again, to our knowledge), the first study to include data for multiple diseases and more detailed time periods of infant lives, while other meta-analyses have predominantly considered one disorder and focused on either pre-natal or post-natal exposure. The broader collection of studies and inclusion of more variables allowed us a more complete overview, allowing more statistical power and more precise estimates of the extent of the effects.

### The double-edged sword of antibiotics

Modern day human societies rely on antibiotics and while they have saved millions of lives, their use is not without cost, as evident from the widespread evolution of antibiotics resistance, in additional to generally poorly understood impacts on health such as those documented here. The antibiotic resistance crisis itself should warrant changing prescription behavior, but according to the WHO we are far from doing so [104]. The confirmative nature of our result support the need to rethink current prescription and individual behaviors to reduce antibiotics use and choose narrow-spectrum antibiotics whenever possible. Notably, although narrow-spectrum antibiotics are preferable, their repeated use may have strong impacts and result in similar disruptive effects [105].

Alternative viable strategies to broad-spectrum antibiotics that can better target bacterial pathogens are needed, and indeed receive substantial attention. One such strategy is phage therapy that relies on the high specificity of bacteriophages to target specific bacteria, principally without harming the commensals in the community [106]. Another novel approach to circumvent permeability-mediated drug resistance is the so-called ‘Trojan horse’, which consists of engineering antimicrobials linked to siderophores—essential iron uptake machinery in bacteria—that can be delivered inside cells to improve uptake, reduce exposure of non-targets and reduce mutation risks [107]. Antimicrobial peptides derived from plants or animals have also been proposed as safer and less resistance-prone alternatives, with their rapid action and recognition of multiple targets slowing down resistance evolution [108]. As progress continues in the development and implementation of such novel approaches to treat infectious disease, so should work to understand if they can alleviate the negative effects of traditional antibiotics on our resident microbes.

### The relevance of the evolutionary perspective

Human evolutionary history transitioned rapidly into modernity, but the pool of genetic, behavioral and physiological traits selected for during the adaptation to our ancestral environments remain. The rapid cultural transition has not been followed by the same rate of evolution, with phenotypic traits such as our ancestral immune physiology adapting slowly, causing mismatches to modernity [109]. This is evident from the well-established consequences of spreading good hygiene practices in modern and contemporary societies [110]. Proper hand washing revolutionized everyday life but only later did it become apparent as an evolutionary mismatch with potential negative consequences [111]. Increased hygiene in households coincided with decreased opportunities for contact with environmental bacteria from the onset of infancy, which is fundamental for immune system training and maturation [112]. Similarly, antibiotics administration decreases the beneficial impact of commensal microbes by affecting their diversity and community composition, especially during vulnerable periods of early childhood. The reduced diversity may change microbial functions that become evolutionary novelties that are either momentary or permanent, in both cases with the potential to affect host physiology long-term. Hygiene practices and antibiotic use may thus shape long-term associations and ultimately our future evolutionary history with symbionts. This is not a first in human microbiome evolution, as the shift from a hunter-gathered to a modern lifestyle already substantially altered our commensal microbiota [113]. It is this ‘new’ microbiota that is now challenged with yet another novelty that shapes its present evolution with tangible impacts and largely unknown future consequences.

Predicting the consequences of a disturbance on a stable symbiosis is complicated: many changes happen over time that would be overlooked when focusing only on the short life-span of individual hosts. It is thus essential to integrate an evolutionary perspective in medical practices that also considers the long history of symbioses when evaluating the impacts of antibiotics on infants’ later health. Evolutionary thinking provides added value when approaching biological problems, and can thus be of help in delivering more effective medical treatments. Concretely, evolutionary theory predicts that resistance to antibiotics will inevitably arise, and evolutionary thinking can provide strategies for measures to delay resistance. It would thus be imperative to train future doctors in evolutionary thinking to, e.g. improve prescription behaviors: antibiotics should not be prescribed during thedelicate period between birth and 6 months unless absolutely necessary. And if antibiotics are unavoidable, they should be as narrow-spectrum as possible and ideally be followed by probiotic treatment to compensate for perturbations to the beneficial microbiota. Taking such approaches may reduce the impact of antibiotics on the microbiota short-term and lifestyle diseases long-term. Finally, integrating even more the evolutionary thinking in medical research could prove essential to allow the shift from cure- to prevention-based practices, both for the presently discussed and other disorders.

### Areas in need of further work: correlation or causality?

The larger number of studies on asthma provided us an overall stronger dataset, more reliable conclusions and the opportunity to test more specific and thus more relevant questions. In contrast, despite finding an overall strong effect of antibiotics use on allergies, the data currently available preclude similar sub-divisions, hence limiting the extent to which we can pinpoint where the strongest effects occur. The same is the case for metabolic disorders: in general, many studies consider impacts on childhood adiposity, but subdivision between obesity, overweight and increased BMI disperses the data and reduces comparative power. This implies that more work to elucidate association is needed if we are to gain a more complete picture of where effects exist and have the largest impact; ideally with more comparable study designs. This will be essential to firmly confirm associations and, more importantly, will help reduce the very high heterogeneity of studies that can currently be compared.

Although antibiotics can cause dysbiosis, establishing the causal links between these microbiota changes and atopic and metabolic disorders is almost universally missing. All epidemiological studies, both prospective and retrospective, remain mainly observational; with the exceptions being Korpela *et al*. [31, 33]. This is unsurprising given the limitations for causation studies in humans, but we should lean on and expand studies in animals, where strong evidence linking microbiota composition to, e.g. obesity [114] have been confirmed after fecal transplants of obese human twins to germ-free mice [115]. Similarly, the critical role of the gut microbiota in immune development has been documented in germ-free animal models that exhibit significant alterations in their immune system which can only be partially rescued by colonization with a normal microbiota [116].

Work to establish the short- and long-term compositional changes caused by exposure to antibiotics have found that it is rather the overall reshaping of the communities than depletion of specific microbes that is responsible for the emergence of disease states after exposure has ceased [117]. Early antibiotic exposure can reduce the diversity and alter the microbial composition for long periods of time [118], and the inclusion of microbiota analysis before, during and after antibiotics exposure would allow linking both overall microbiota changes and effects on specific commensals, with the pathology of disorders. If causality can be established, reevaluating antibiotic types and integrating probiotics administration could help reduce the risk of disease emergence after treatment of infections where antibiotics are unavoidable.

The present study focused on the impact of antibiotics use early in life on autoimmune and metabolic disorders; both being major disorders with known links to microbiota disturbances [119, 120]. However, normal microbiome composition and function are associated with a series of other health aspect that may hence be prone to similar negative impacts of microbiota perturbations during infancy. For example, bacteria affect host physiology and normal brain functions through microbial neurometabolites interacting with the neuronal network in the so-called ‘gut-brain axis’ [4]. Dysbiosis may thus contribute to the development of behavioral and neurological disorders [121]. Indeed, microbiota composition has been linked to increased stress and anxiety [122] and may also play a role in the surge of neurodevelopmental disorders, such as autism spectrum disorders and schizophrenia [123, 124]. Research on the indirect impacts of antibiotics remain few [125], but future meta-analyses may well establish patterns of association as we document for atopies and metabolic disorders. Finally, numerous are the reports of rampant antibiotics use in developing countries, but the consequences of this remain largely unknown.

## CONCLUSIONS

The present meta-analyses provide strong evidence for associations between antibiotic use prenatally and during early infancy with increased risks of autoimmune and metabolic disorders such as asthma and obesity. In particular, we point to the first 6 months of life as the most sensitive period, where even temporary changes in the microbiota may have lasting impacts, and where the number and types of antibiotics courses have strong effects. Our findings support that it is advisable to reduce antibiotics use and implement alternative therapeutic approaches when possible. Related to this, improved knowledge dissemination of risks from the scientific community to the regulatory field and the public is worthwhile. Our and the myriads of species surrounding us are the result of evolution that is not in isolation, but through intricate and numerous associations which shaped our past and will shape our future: the better we understand how our evolutionary history is intertwined with symbionts and how our modern mismatched living negatively impacts their well-being, the better can we target treatment to improve public health and understand how symbionts affect human evolution.

## SUPPLEMENTARY DATA

Supplementary data is available at *EMPH* online.

## Supplementary Material

eoaa039_Supplementary_Data
