# Supplementary material for: The impact of early life antibiotic use on atopic and metabolic disorders: Meta-analyses of recent insights
Source: Evol Med Public Health. 2020 Oct 24;2020(1):279–89. doi: 10.1093/emph/eoaa039 (PMC7723877; doi:10.1093/emph/eoaa039)
Supplement: eoaa039_Supplementary_Data [file eoaa039_supplementary_data.zip › Table S4.docx]

Table S4. Summary of overall and subgroup – when available – meta-analysis conducted on studies associating early antibiotic exposure to metabolic disorders.

| **Outcome** | **Study** | **Pooled estimate (95% CI)** | **p-value** | **I2** |
| --- | --- | --- | --- | --- |
| **Obesity** |  |  |  |  |
| Total studies | 15 | 1.14 (1.07, 1.23) | 0.0002 | 92% |
| Prenatal exposure | 6 | 1.12 (1.02, 1.25) | 0.0004 | 78% |
| Infancy exposure | 7 | 1.16 (1.03, 1.30) | <0.00001 | 96% |
| Exposure to BS antibiotics | 4 | 1.11 (1.07, 1.16) | <0.00001 | 76% |
| **Overweight** |  |  |  |  |
| Total studies | 11 | 1.11 (1.05, 1.17) | <0.0001 | 72% |
| Prenatal exposure | 4 | 1.11 (1.04, 1.18) | 0.001 | 36% |
| Infancy exposure | 8 | 1.19 (1.05, 1.34) | 0.005 | 74% |
| **BMI-z score** |  |  |  |  |
| Total studies | 12 | 0.06 (0.03, 0.08) | <0.00001 | 80% |
| Prenatal exposure | 4 | 0.04 (0.00, 0.07) | 0.05 | 84% |
| Infancy exposure | 9 | 0.08 (0.04, 0.12) | <0.00001 | 71% |
