# Supplementary material for: The impact of early life antibiotic use on atopic and metabolic disorders: Meta-analyses of recent insights
Source: Evol Med Public Health. 2020 Oct 24;2020(1):279–89. doi: 10.1093/emph/eoaa039 (PMC7723877; doi:10.1093/emph/eoaa039)
Supplement: eoaa039_Supplementary_Data [file eoaa039_supplementary_data.zip › Table S3.docx]

Table S3. Summary of overall and subgroup – when available – meta-analysis conducted on studies associating early antibiotic exposure to atopic disorders.

| **Disorders and comparisons** | **Number of studies** | **Pooled ORs**  **(95% CI)** | **p** | **I2** |
| --- | --- | --- | --- | --- |
| **Asthma** |  |  |  |  |
| Total studies | 28 | 1.67 (1.47, 1.89) | <0.0001 | 99% |
| Prospective cohort studies | 16 | 1.58 (1.35, 1.85) | <0.0001 | 98% |
| Retrospective cohort studies | 11 | 1.71 (1.49, 2.10) | <0.0001 | 99% |
| Prenatal exposure | 9 | 1.24 (1.21, 1.28) | <0.0001 | 47% |
| Infancy exposure (0-6 months) | 5 | 1.80 (1.45, 2.23) | <0.0001 | 45% |
| Infancy exposure (6-12 months) | 17 | 1.76 (1.45, 2.14) | <0.0001 | 99% |
| Infancy exposure (12-24 months) | 8 | 1.40 (1.30, 1.51) | <0.0001 | 67% |
| Exposure to broad-spectrum AB | 5 | 1.34 (1.12, 1.60) | 0.001 | 92% |
| **Eczema** | 9 | 1.41 (1.18, 1.69) | <0.0001 | 81% |
| **Allergies** | 7 | 1.51 (1.39, 1.64) | <0.0001 | 6% |
