# Supplementary figures and images for: The impact of early life antibiotic use on atopic and metabolic disorders: Meta-analyses of recent insights
Source: Evol Med Public Health. 2020 Oct 24;2020(1):279–89. doi: 10.1093/emph/eoaa039 (PMC7723877; doi:10.1093/emph/eoaa039)

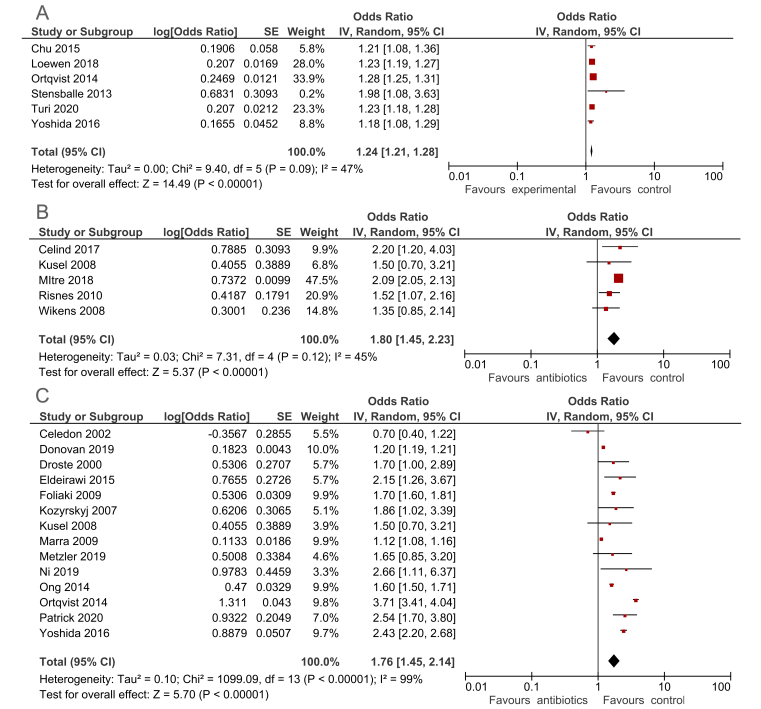

Supplement: eoaa039_Supplementary_Data [file eoaa039_supplementary_data.zip › Figure S1.1.tif]

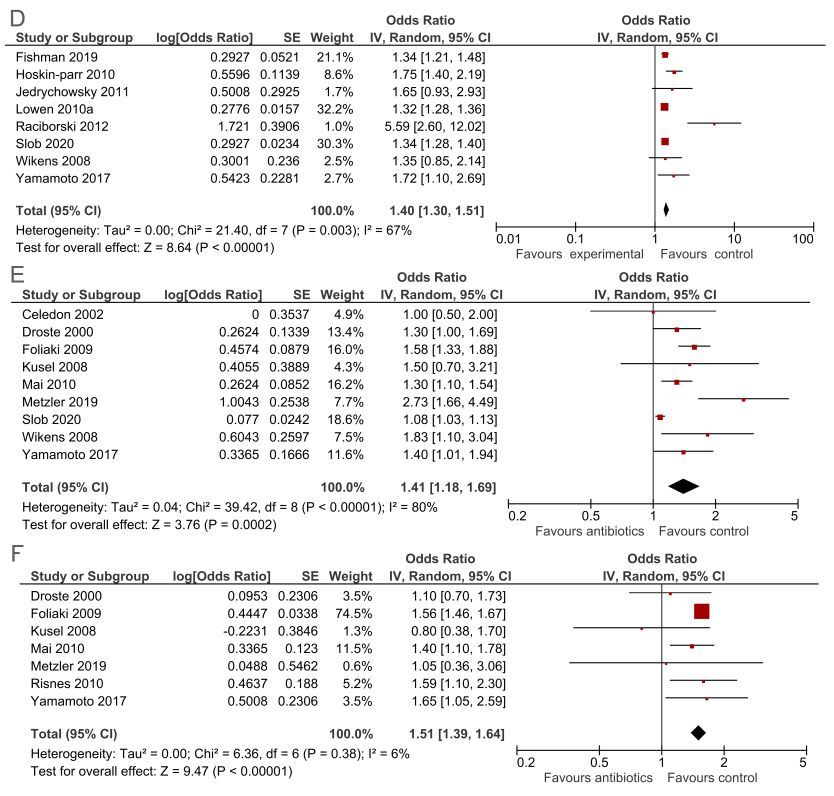

Supplement: eoaa039_Supplementary_Data [file eoaa039_supplementary_data.zip › Figure S1.2.tif]

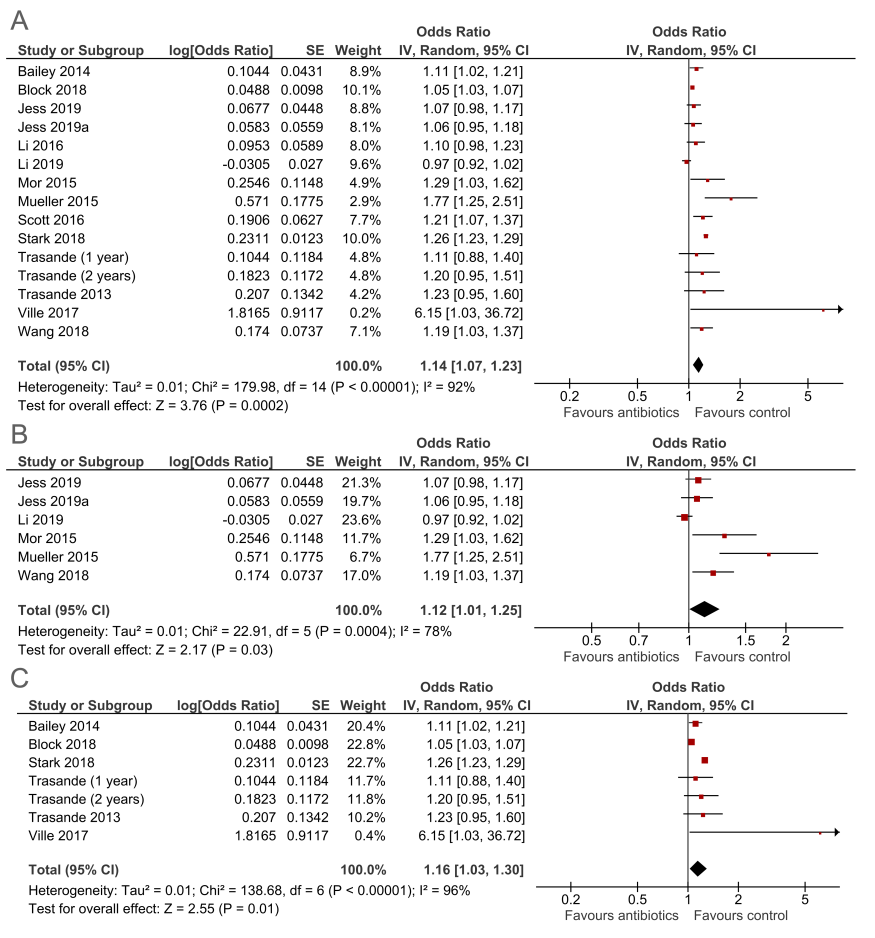

Supplement: eoaa039_Supplementary_Data [file eoaa039_supplementary_data.zip › Figure S2.tif]

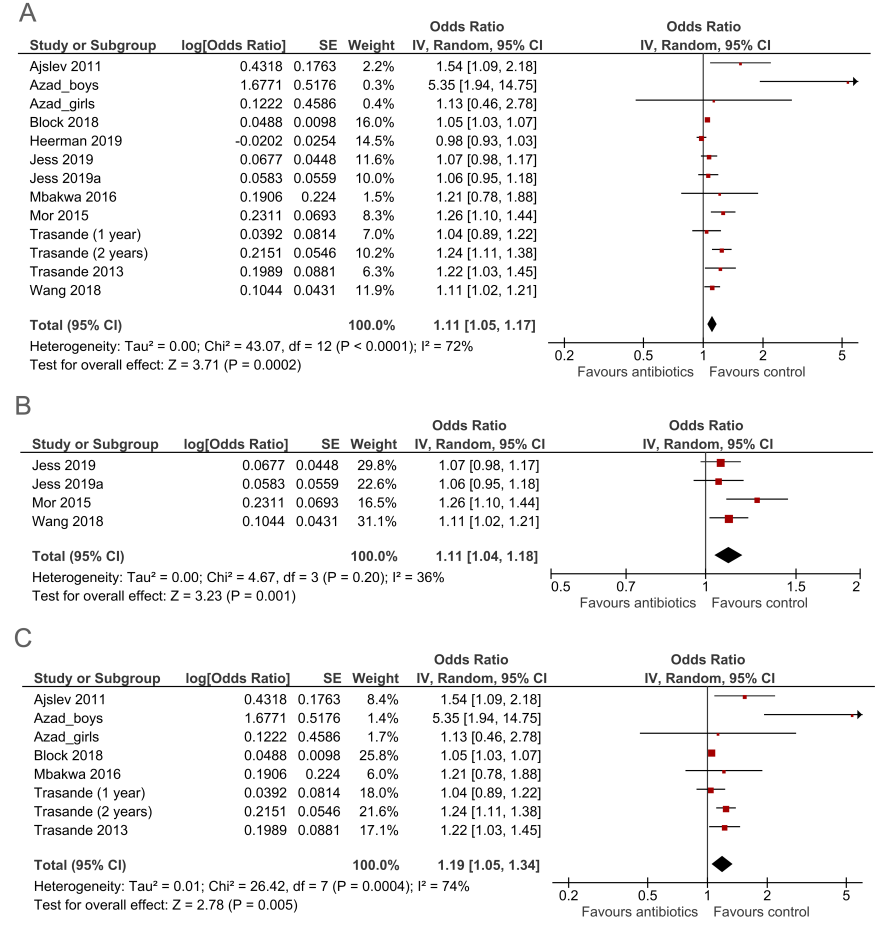

Supplement: eoaa039_Supplementary_Data [file eoaa039_supplementary_data.zip › Figure S3.tif]
